# Deep Learning-Based Cell-Level and Beam-Level Mobility Management System †

**DOI:** 10.3390/s20247124

**Published:** 2020-12-11

**Authors:** Roman Klus, Lucie Klus, Dmitrii Solomitckii, Jukka Talvitie, Mikko Valkama

**Affiliations:** 1 Electrical Engineering Unit, Tampere University, 33014 Tampere, Finland; lucie.klus@tuni.fi (L.K.); dmitrii.solomitckii@tuni.fi (D.S.); jukka.talvitie@tuni.fi (J.T.); mikko.valkama@tuni.fi (M.V.); 2 Institute of New Imaging Technologies, Universitat Jaume I, 12071 Castellón, Spain

**Keywords:** 5G New Radio, artificial neural network, beam-level mobility, handover, mobility management, supervised learning

## Abstract

The deployment with beamforming-capable base stations in 5G New Radio (NR) requires an efficient mobility management system to reliably operate with minimum effort and interruption. In this work, we propose two artificial neural network models to optimize the cell-level and beam-level mobility management. Both models consist of convolutional, as well as dense, layer blocks. Based on current and past received power measurements, as well as positioning information, they choose the optimum serving cell and serving beam, respectively. The obtained results show that the proposed cell-level mobility model is able to sustain a strong serving cell and reduce the number of handovers by up to 94.4% compared to the benchmark solution when the uncertainty (representing shadowing, interference, etc.) is introduced to the received signal strength measurements. The proposed beam-level mobility management model is able to proactively choose and sustain the strongest serving beam, even when high uncertainty is introduced to the measurements.

## 1. Introduction

The 5G New Radio (NR) networks introduce new challenges to mobility management due to tightened beamforming requirements. In the previous generations of networks, each base station covered a relatively wide area, and the mobility management performed a handover (HO) only when the User Equipment (UE) left the serving area, or when the network performed load-balancing procedure (e.g., cell breathing in 3G). In beamforming-enabled networks, each base station deploys a number of beams, each covering only a small area. As a result, mobile UE frequently changes serving beam, and the network mobility management has to perform beam-level mobility operations reliably and with minimum delay. Delayed beam switching in the mobile scenario may cause a significant decrease in throughput and, in the worst case, a loss of the radio link. Beamforming-capable base stations were introduced in later releases of Long Term Evolution (LTE) networks with only a few beams available. In NR, mostly in frequency band 2, the number of beams deployed from a single base station (BS) is increased to up to 64 [1].

In this paper, we address the challenge of cell-level and beam-level mobility management while utilizing a supervised learning approach, namely Artificial Neural Network (ANN or NN) architecture, to handle cell-level and beam-level mobility separately. The proposed solution performs beam and cell selection on a BS side from the reported Reference Signal Received Power (RSRP) and positioning information by the UE. The Machine Learning (ML) solution enables the model to be purely data-driven. The same proposed model for cell-level and beam-level mobility management can be used on any BS and perform reliably after being properly trained. The data utilized in this work, including RSRP measurements and positioning information, come from simulated scenario in urban deployment. The goal of our work is to (1) reduce the number of unnecessary HOs while (2) sustaining highest possible RSRP level. We introduce different levels of uncertainty to the RSRP measurements to simulate real-world distortion of the measured data and compare the performance of the proposed ML model to the benchmark 3rd Generation Partnership Project (3GPP) model.

The need for proactive mobility decision-making in 5G networks, which supports high user mobility, beamforming-capable BSs and low tolerance for errors, can be addressed by utilizing ML. The solutions that satisfied the requirements in the previous network generations, such as BS ranking in 2G or the 3GPP model utilized in 4G networks, have either too large delays and interruption times, or are not resilient enough to the dynamics of the environment caused by the strong attenuation of the signal at higher frequencies, among other factors. Addressing cell-level and beam-level handovers as separate challenges is the essential point of this work. In dense network deployments, beams from separate BSs are likely overlapping. UE moving through the beam overlapping area may be forced to perform cell-level handovers instead of simply switching to neighboring beam from the same BS, putting unnecessary load on the network due to increased control and signaling overhead. Thus, reducing the number of inter-cell handovers is the additional goal of this solution.

As a result of our work, we obtained the optimized beam-level mobility management per each beamforming-capable BS, as well as optimized cell-level mobility management, distributing users between the individual base stations. To achieve that, we utilized ANN architecture in mm-Wave deployment, which, to our best knowledge, was not addressed in any other work so far.

### 1.1. Main Contributions

In this work, we study an ML-based solution to enhance the performance of 5G NR mobility management in terms of cell-level and beam-level mobility. We focus on modern approaches in the Deep Learning (DL), utilizing convolutional layer architecture. The main contributions of this work are summarized as follows:

1. We propose a novel solution with two ANN models for HO management considering both cell-level and beam-level mobility as separate challenges.
2. To apply the model to a real-world scenario, we propose a 3-stage implementation, including data-mining, validation, and operational phases.
3. We evaluate the performance of the proposed models and show improved performance related to the number of required HOs and the observed link quality when comparing to the benchmark 3GPP solution.

The rest of the paper is organized as follows. The rest of Section 1 includes the related work review. Section 2 presents the essential background information, including mobility management in 5G NR, data acquisition, performance metrics, measurement uncertainties, and machine learning principles. Section 3 contains information about the proposed solution and its application in practical scenarios. Moreover, Section 4 presents evaluation environment utilized in this paper, numerical results of the proposed solution, and a short discussion about possible improvements and challenges, and, finally, Section 5 concludes the work.

### 1.2. Related Work

The presented related work focuses on two topics, namely mobility management solutions in 5G NR as the area of interest and machine learning paradigms as the chosen solution.

This work extends the idea of Reference [2], in which the authors proposed a single ANN to handle HO management in an urban scenario. The model managed HOs across 7 BS, each with 32 possible beams, containing both convolutional and dense architectures. The results of the analysis show a significant decrease in the number of HOs at higher uncertainty levels while sustaining a high RSRP link. The paper does not distinguish between cell-level and beam-level mobility.

The topic of artificial intelligence (AI) in modern radio networks was addressed in multiple research papers. Yao et al. [3] discuss the key enablers of 5G radio networks and corresponding AI opportunities. Massive Multiple Input Multiple Output (M-MIMO) systems, spectrum management and network densification are highlighted as primary areas of interest. The presented AI solutions include sequential and recurrent architectures in the physical layer or configurable models for AI-aided 5G BS. The work highlights the challenges and limitations of current AI regarding computational requirements, required data quality or security. Surveys [4,5,6,7,8] discuss the machine learning paradigms in modern radio networks, addressing history, key challenges, and limitations of the current state of the art. O’Shea et al. [9] present the possibilities of DL for the physical layer. The surveys focused on the reinforcement learning applications in the radio networks include [10,11]. Next, the survey on LTE and 5G NR HO management [12] presents the in-depth overview of the currently used procedures, requirements, and concepts of the HO management. The work highlights the key differences between the 4G and 5G radio networks and provides an exhaustive review of the related literature.

In Reference [13], the authors propose an ML solution to enable coordinated beamforming by mapping the received uplink pilot signals. The results show the improved throughput when compared to the benchmark model, as well as high reliability. Wang et al. [14] propose a HO optimization system for IoT devices, while utilizing multiple ML techniques including clustering of users based on their paths, deep reinforcement learning to optimize HO decision-making based on the user’s cluster and supervised learning to pre-train the utilized NNs. The paper shows substantial performance improvement in traditional networks. The same research group proposed a Deep-Q-learning approach in the ultra-dense scenario to optimize the HO decision-making of the UE [15]. The UE downloads the weights of the pre-trained model at arrival to the area of interest, then updates the weights based on its behavior in reinforcement learning fashion. The downside of this work is the model training, which is performed on the UE side, draining battery life due to computational requirements of the training procedure. Shen et al. [16] utilize a ϵ-greedy bandit algorithm to optimize the HO decision-making. The work proposes an additional constant C, which is added to the reinforcement learning loss when performing a handover. The proposed solution outperforms a benchmark LTE 3GPP model by reducing the number of HOs by 80%. Shi et al [17] propose a Lagrange interpolation model to reduce the number of unnecessary HOs. Their mobility prediction model achieved up to 90% prediction accuracy while decreasing the number of HOs by up to 30%. Chen et al. [18] utilize software vector machine (SVM) classifier to predict user’s next serving base station, in order to improve resource allocation of the system, achieving very high accuracy in traditional networks. Joud et al. [19] utilize user clustering in vehicular and highly mobile networks to improve the throughput of the network with positive results. Zhang et al. [20] achieved 40% HO decrease over traditional 3GPP LTE scheme by calculating HO probabilities using stochastic geometry analysis in the user-centric network. Viering et al. [21] investigate the 0 ms interruption soft handover systems, which aim to eliminate the HO interruption time.

The summary of the research work presented above shows the promising results achieved by other research groups in improving mobility management using machine learning techniques. Despite that, none of the above focus directly on the implementation, which complies with 3GPP standards. Ensuring the interoperability of any proposed solution with the existing systems and within standards is today crucial, since the new generations of networks increase in complexity and in requirements on each network component. The related work also fails to address the mobility management as a whole, while distinguishing between cell-level and beam-level mobility. The system proposed in this work is compatible with the newest 3GPP standards, while its inputs are user data available at the BS during the network operation. We optimized the proposed solution to operate with minimum delay by reducing the size of the NNs and by segmenting the mobility management task into two separate challenges, namely cell-level mobility and beam-level mobility optimization.

## 2. Preliminary Information and Scope

In this section, we present how does the proposed solution comply with current systems and standards, as well as briefly discuss the necessary background and scope of this work.

### 2.1. Mobility Management in 5G

The core of mobility management in 5G NR is very similar to the one in 4G LTE, which is discussed in detail in Reference [22]. As specified by 3GPP Rel. 15 [23], mobility in mobile networks is divided into Radio Resource Control (RRC)_IDLE mobility, RRC_INACTIVE mobility, and RRC_CONNECTED mobility. RRC_IDLE and RRC_INACTIVE are states in which UE does not have any established RRC connection. RRC_CONNECTED means that UE has an active RRC connection to the network and, therefore, is ready to receive or transmit user-plane data. In case UE changes its location (or triggers other event leading to mobility management action) in RRC_CONNECTED state, the network changes means of communication with the UE.

**Figure 1:**
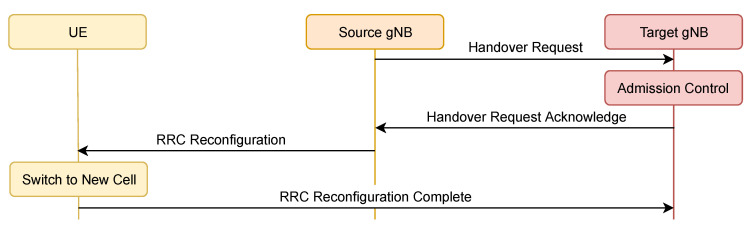
INTER-gNB Xn handover procedure.

Cell-level mobility or handover refers to transferring the ongoing data transmission from one network component (gNodeB (gNB), Remote Radio Head (RRH), etc.) to another. The handover procedure consumes resources, causes connection interruption and differs depending on the handover type (INTER/INTRA-gNB, with or without 5G Core Access and Mobility Management Function (AMF) change, etc.), the network requires a varying level of internal communication and signaling, where the least complex one, INTER-gNB Xn handover procedure [12] is depicted in Figure 1.

The INTER-gNB Xn handover procedure includes four components.

- Based on the received measurements, Source gNB initiates the handover through Xn interface.
- Target gNB provides new RRC configuration and performs admission control.
- Source gNB forwards the Handover Request Acknowledge message to the UE, along with cell ID, access information and beam-specific information.
- UE moves to RRC connected state with Target gNB and sends the RRC Reconfiguration Complete message.

Xn handover does not include AMF and User Plane Function (UPF) in the handover procedure. In case they are included (e.g., in Next-Generation (NG) handover), the procedure consists of additional steps, further increasing the complexity and the delay required by the process. Radio-frequency resources, as well as processing power, are consumed with each handover, which makes minimizing the number of unnecessary handovers a key requirement of an efficient mobility management system. The frequency of inter-cell handovers is one of the critical parameters that influence the efficiency of mobility management, along with the high signal to interference and noise ratio (SINR) communication.

Beam-level mobility refers to the mobility management event, in which the beamforming-capable antenna array of a single gNB changes only the beam created by the same antenna array. This type of mobility does not require any network-level signaling and is resolved locally at the physical layer and Media Access Control (MAC) layer only [12] to maximize the speed and efficiency of the operation, as well as since there is no need for the upper layers to take part in this particular decision-making. This kind of mobility events occur much more frequently than cell-level events, and erroneous beam-level decisions may lead to decreased Quality of Experience (QoE) (from user’s point of view) and Quality of Service (QoS) (from network’s point of view).

To enable beam-level mobility, gNB defines the necessary measurement configuration for the UE, including structure for Secondary Synchronization Block (SSB)/Channel State Information (CSI) reporting, trigger states, and interference measurements. The BS receives information about the channel state (including RSRP) through SSB CSI. Trigger states define the means and frequency, by which the CSI measurement is obtained and transmitted at the UE side. Interference measurements define the ways to measure channel parameters, such as RSRP. The mobility itself is then handled based on the defined configurations without RRC’s knowledge about the current beam configuration. There is a significant difference between handovers and beam-switching actions, as the latter does not require any network-level signaling. Therefore, while considering beam-level mobility, reducing the frequency of beam-switching is a secondary optimization goal, while sustaining high quality connection at all times is the primary objective.

### 2.2. Network Data Acquisition

The network measurement considered in this work is RSRP, which, in NR networks, is divided into two separately measured entities [1].

Synchronization signal (SS) RSRP is measured across different frequencies, even outside the current operating band. It is calculated from the secondary synchronization signal (SSS), as the linear average over the resource element’s power contributions. SSS is transmitted periodically, as a part of the SSB transmitted by the BS. This work considers the measured RSRP values as SS RSRP, as all available beams are considered at an equal level.

CSI RSRP is measured only in the currently operating band, as a part of the CSI reference signal. SSB is transmitted in SSB sets by each BS over each deployed beam in 5 to 160 ms intervals. The default interval specified by 3GPP is 20 ms, which is considered as an interval in which a single UE is able to obtain a new RSRP measurement from each beam and each BS. Maximum number of SSBs per a SSB set is 64 for 5G frequency band 2, which complies with BSs considered in this work, each deploying 32 beams.

UE sends the measured RSRP information to the serving BS and the network in CSI reports, as a part of the physical uplink control channel (PUCCH). CSI reporting can be periodic, defined by the serving BS, or aperiodic, which is event-triggered and also determined by serving BS. The combination of periodic and aperiodic CSI reporting is possible using the semi-persistent setting.

### 2.3. Mobility Performance Metrics

In order to evaluate the health and efficiency of mobility management, numerous HO performance metrics were presented in the literature [12,24,25], reflecting different aspects of the handover success or failure. In the scope of this work, we utilize the following metrics.

- HO count represents the number of HOs performed by the model on the considered dataset. In the literature, a more common evaluation metric is HO rate (or HO frequency) calculated as the multiplicative inverse of the mean number of measurements between two HO events. HO count was chosen as the considered metric, as it directly reflects the load on the system caused by HOs. We consider HO count as a metric for both cell-level and beam-level mobility events. HO count is directly proportional to HO energy consumption considered in Reference [12].
- Serving RSRP represents the signal quality of the selected serving beam at every measured instance. We also evaluate mean RSRP as the average of the serving RSRPs on the whole considered dataset. Maximizing the serving RSRP maximizes the possible throughput of the telecommunication system.

### 2.4. RSS Measurement Uncertainties

One of the goals of the proposed solution is to cope with different levels of measurement uncertainty. Measurement uncertainty is affected by a combination of deterministic and stochastic aspects [26]. The deterministic ones can be measured and directly included in the system analysis, whereas stochastic ones are random variables changing over time. Sources of measurement uncertainties include hardware imperfections, dynamic changes in the channel due to movement, and interference from other sources. NN models can cope with both deterministic and stochastic uncertainties, as well as both are included in the training set, and the model is powerful enough.

The uncertainties in the RSS measurements can be characterized using normal or log-normal random variable [27]. In the scope of this work, we model the uncertainty as the normal distributed random variable added directly to the dB-scale RSRP measurements as $N(0,\sigma^{2})$, where σ denotes standard deviation and, in this work, varies between 0 dB and 5 dB. Normal distribution was chosen over log-normal, since log-normal distribution is always larger than 0, while many uncertainty aspects (e.g., shadowing) decrease the received signal strength. As the reference for the chosen range, we considered that 3 dB attenuation refers to the signal strength reduction by 50%, as well as the 3 dB default hysteresis margin value in LTE networks.

### 2.5. Machine Learning and Neural Networks

ML is a discipline of data science, in which the goal is to extract useful information from data without being explicitly programmed for that task [28]. NNs are ML solutions, which are based on the functionality of the human brain. The NN models consist of numerous interconnected layers of neurons, which sequentially process the information (in most cases). We chose NN architecture due to its ability to adapt a single model to multiple scenarios, its off-the-shelf performance in classification tasks and its property to modify the size of the model by adding, subtracting, or changing layers to optimize its performance. ML also enables proactive, rather than reactive, decision-making of the chosen solution.

In this work, we utilized the NN architecture with two kinds of layers, convolutional layer and dense layer. The dense layers are most commonly utilized layers, in which each neuron from the previous layer is connected with every neuron of the current layer. This results in a high number of trainable parameters and therefore, high training complexity of the model. On the other hand, dense layers can solve tasks universally. The convolutional layer is frequently used in machine vision tasks due to its feature extraction properties, the low number of trainable parameters and the ability to stack multiple convolutional layers in a row. The disadvantage of convolutional layers lies in the issue that they do not work with all kinds of data. We utilized hybrid NN architecture, consisting of convolutional feature extractor and dense core network to address the mobility management challenge.

## 3. Proposed Solution and Operation

In this paper, we propose a novel, DL-based solution for beam-level mobility management in the scope of 5G NR, with positioning information included in the model for higher stability. We supplement the beam-level mobility model with overlaying, HO management model, responsible for user mobility on the cell level.

### 3.1. System Model

**Figure 2:**
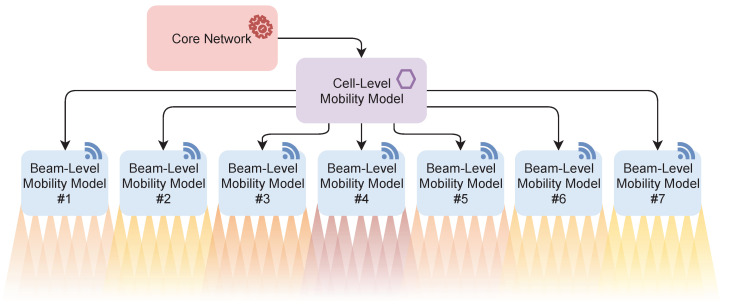
Proposed hierarchical system model.

The system considered in this paper is presented in Figure 2. In order to satisfy the optimal mobility decision-making and reduce the number of unnecessary inter-cell HOs, the proposed DL-based system is divided into the following parts.

**Figure 3:**
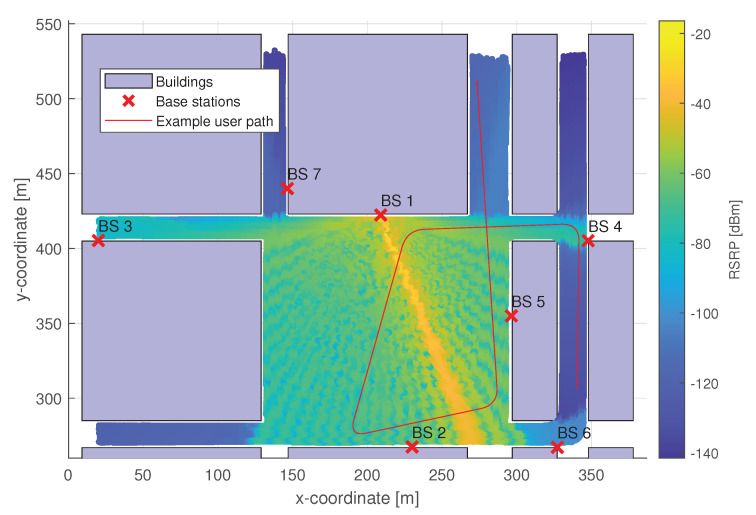
The considered network deployment with highlighted buildings, base station (BS) positions, example user path, and an example coverage of a single beam.

- Each base station (BS1 to BS7 in Figure 3) is operated by a single beam-level mobility model, which is trained to choose the optimal serving beam (out of 32 available beams). The strongest candidate beam determines the optimal target beam. Each BS operates independently and operates only when the cell-level mobility model assigns it to the user (see Figure 2).
- A single-cell level mobility model is handling the HO decision-making between individual BSs belonging to the given area. There is no beam-specific information available in this model, only the BS-specific information sent by each of the BSs. The base station-specific information is determined by the strongest available beam of that BS, and the HO decision-making is trained on optimal 3GPP model labels with hysteresis margin of 3 dB.

To summarize, the core network assigns users to the cell-level mobility model (e.g., via inter-gNB handover with AMF change). The cell-level mobility model then assigns the users between individual BSs (gNBs). On each BS, the beam-level mobility management model assigns each user the serving beam. The core network only knows the approximate user location (area level information), the mobility management model has serving cell information, and the serving beam information is only available at the serving beam-level mobility model level.

### 3.2. Benchmark 3GPP Model

As a benchmark model for the cell-level, as well as the beam-level mobility, a 3GPP model [29] is utilized. The behavior of the 3GPP model is defined by the hysteresis margin (3 dB by default), which specifies the required difference between the serving and candidate cell or beam RSRP values to initiate a handover. Specifically, the 3GPP model operates per sample (Algorithm 1) as follows:

**Algorithm 1** The 3GPP Model Per-Sample Algorithm.  $serving\_index_{1}=i$  **for**
t=1:T
**do**   $j=argmax(RSRP^{t})$   **if**
$RSRP_{i}^{t}+HM\leq RSRP_{j}^{t}$
**then**    $serving\_index_{t+1}=j$   **end if**  **end for**

$serving\_index_{t}$ denotes the serving cell (or beam) index at time instance *t*, $RSRP^{t}$ represents the vector of all RSRP measurements at time instance *t*, $RSRP_{i}^{t}$ is the RSRP value of the *i*th cell (or beam) in *t*th time instance, and HM is the hysteresis margin. The RSRP of the candidate cell or beam *j* has to be larger than the RSRP of the serving cell or beam *i* at least by the hysteresis margin HM in order for the system to initiate a handover.

This model is used in LTE networks and serves as the benchmark model in the referred literature [2,15,16]. This benchmark model is purely reactive, as it can react to the environment-specific signal changes with a delay of at least 1 measurement period. It is also susceptible to any measurement uncertainties, which cause the model’s performance to quickly degrade as shown earlier in Reference [2]. Due to this fact, the benchmark 3GPP model’s decision-making may result in numerous ping-pong HOs.

The hysteresis margin of the 3GPP model defines the trade-off between always sustaining the highest RSRP connection and the number of performed HOs. Lowering the hysteresis margin also makes the system more vulnerable to the effects of measurement uncertainties, as smaller peaks in RSRP measurement can cause the system to perform an unnecessary HO. The trade-off is depicted in Table 1 for varying hysteresis margin levels for BS1 data with 0 dB uncertainty. The effect of increasing uncertainty level is shown in Section 4.2. In this work, the 3GPP model with hysteresis margin of 3 dB was chosen as the benchmark solution since it is a default hysteresis value in the standards, and due to the reasonable trade-off ratio.

### 3.3. Beam-Level Mobility Model

The basis of the beam-level mobility model proposed in this work is similar to the one proposed in Reference [2]. Instead of providing mobility over the whole area covered by multiple base stations and their beams, it operates on a single base station for beam-level mobility only. The following adjustments were made to optimize the model’s performance and remove redundant information fed to the DL model. The dimensions of the model were reduced when compared to the solution from Reference [2], as it now serves as 33-target classifier with the reduced number of inputs, and one of the input parameters, namely the serving beam information, was also removed from the model. The number of targets is determined by the number of beams that the BS is able to transmit (32) plus one, indicating that no beam is suitable (lack of BS signal detection by UE). Reducing the size of the model enables the model itself to perform and train faster in the considered environment and improves the optimization and debugging process, as well as reduces the overall complexity of the system as a whole. Additionally, the operation of the system is more resistant to the single-point-failures due to the higher number of independent elements in the system (multiple smaller models instead of a single, large one).

**Figure 4:**
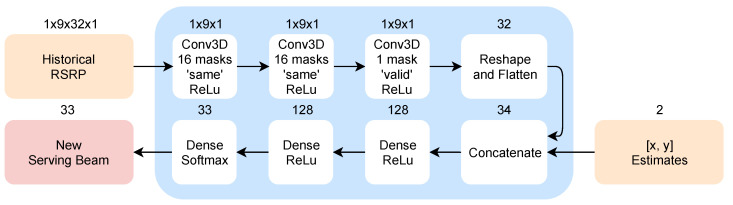
Proposed beam-level mobility model architecture.

The structure of the proposed beam-level mobility model is shown in Figure 4. Its first input, including current and 8 last RSS measurements per each beam, is processed by the convolutional branch of the model. The window size of 9 measurements was derived experimentally. Larder window sizes did not improve the model’s performance and increased the complexity of the proposed solution, while the performance of the model decreased with smaller window sizes, after introducing uncertainty to the measurements. The convolutional branch includes 3 convolutional layers and serves as the feature extractor for the next part of the model, as well as a signal filter. All three convolutional layers have a filter shape of 1×9×1, Rectified Linear Unit (ReLU) activation function, ‘same’ padding, and 16 masks, with the last layer having ‘valid’ padding and 1 mask. Its result is then concatenated with the second input, namely current positioning information. As stated above, the current serving beam information was removed from the model due to the fact, that the proposed beam-level mobility model does not consider hysteresis. The beam-switching stability of the model is ensured in the training phase. Additionally, beam-level decision-making does not require additional network signaling and works with much lower interruption times than normal HO. The rest of the model consists of three dense layers, with new serving beam information evaluated at the output. The model was designed to minimize the complexity of the system and speed up both training and evaluation, and, as a result, it contains only 27,874 trainable parameters.

Supervised learning approach requires the initial (training) dataset to be available for the location of deployment. The labels determining the ground truth for the *i*th sample for the training of the beam-level mobility model is calculated as follows. The vector of all 32 beam strength measurements for samples i−2 to i+8 (11×32 matrix) is multiplied by a weight vector w=[0.5,0.75,1,1,0.8,0.6,0.4,0.2,0.1,0.1,0.1], which works as a smoothing function for the RSRP values. The resulting vector of weighted beam strengths is then passed through an argmax function, returning the index of the strongest beam. The target is then the vector of zeros with a single one, at the strongest beam’s coordinate. In case all beam measurements are below −90 dBm, the target coordinate is the 33rd index, signaling no beam is available.

### 3.4. Cell-Level Mobility Model

**Figure 5:**
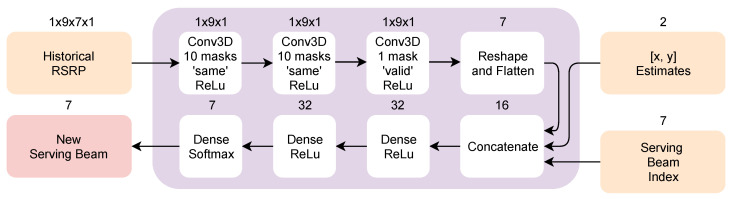
Proposed cell-level mobility model architecture.

The overlaying model, which is designed to handle the cell-level mobility management, was designed to reliably handle the inter-cell mobility management with a reduced number of the required information at the input. Its architecture is depicted in Figure 5. The proposed model contains only 2932 trainable parameters. The number of parameters has been reduced compared to the solution from Reference [2], since the cell-level classifier only chooses between 7 available BS, serving as individual targets for the model, as well as it takes a reduced number of inputs.

Similar to the beam-level mobility model, the proposed solution for cell-level mobility first takes the 8 last reported RSRP measurements from each base station (each representing the current and historical measurements of that base station’s strongest beam) and passes them through the convolutional branch composed of 3 functional layers. All three convolutional layers have a filter shape of 1×9×1, ReLU activation function, ‘same’ padding, and 10 masks, with the last layer having ‘valid’ padding and a single mask. The resulting features are then concatenated with current positioning estimates of the target UE and the current serving beam index, including which is now necessary due to the hysteresis margin for HO decision-making. The following layers are two dense layers with ReLU activation function and 32 neurons each, followed by a dense output layer with 7 neurons and ‘softmax’ activation function. For the cell-level mobility model, the true labels were defined using uncertainty-free 3GPP model labels from the next measurement period. As the result, our cell-level mobility management model predicts the delay-free labels of the 3GPP model with 0 dB uncertainty and 3 dB hysteresis margin, regardless of the uncertainties present at the input.

The benchmark model for the cell-level mobility management is the 3GPP model with hysteresis margin 3 dB, which considers only the strongest beam from each base stations as the input.

### 3.5. Real-World Operation

Gathering the data, based on which the models can be trained is today one of the main challenges of DL. The proposed solution is trained and operates purely on the user behavior in the considered area; therefore, it requires the location-specific dataset in order to properly function. As such, we propose the following solution to enable the usage of the DL models in deployed networks.

1. The first stage of operation focuses on data mining from the users, while operating the network based on the default (e.g., considered 3GPP model) solution. In this stage, all BS are saving all reported measurements along with user locations, creating a robust dataset. The user-based data can contain high levels of uncertainties. A filtering procedure should be applied before using such data for DL model training. This stage can be supported by performing professional measurements at the deployed area, providing unbiased and reliable database, on which the model can set the initial weights (e.g., by pre-training on such data).
2. The second stage, called validation, takes place after the initial model training with the data acquired in the previous stage. The trained AI model operates in parallel with the previously operating default model, which is still responsible for the decision-making. During this stage, the outputs of the proposed solutions are evaluated by the system.
3. The third, operational stage begins when the evaluating system confirms that the training was successful and that the DL model outperforms the default one. The real-world decision-making becomes the DL model’s responsibility, while the default model serves as a backup. In this stage, the data from the users are still continuously gathered and the model is periodically re-trained to be able to cope with changes in the environment (e.g., new building, increased attenuation by trees during spring or other temporary constructs). New, permanent changes in the environment will be trained with the new samples, which contain their effects. Temporary changes in the environment will be “forgotten” when the newly gathered samples will not contain their effects any more. Predicting using the trained model is computationally inexpensive, while training of the model requires many computational resources. The first option to enable the training is to outsource the training procedure to another server (e.g., cloud) and only perform the operation in the network node itself. The second option is to train the model directly at the network node, at the time when the network traffic is minimal, e.g., at night, adjusting the computing resources allocation based on the network usage patterns.

## 4. Numerical Results

In this section, we present the evaluation environment, and the numerical results of our solution at different levels of RSRP uncertainty. We present the results regarding the cell-level mobility model and the beam-level mobility model and their comparison to the benchmark. All models were evaluated on the independent testing set, which was separated from the training and validation data before training.

### 4.1. Evaluation Environment

The considered wireless channel model is based on a ray-tracing-based radio propagation modeling proposed by the Mobile and wireless communications Enablers for the Twenty-twenty Information Society (METIS) project [30], which is included in the 3GPP specification 38.901 [31]. Detailed information about specific radio propagation characteristics and channel model properties are discussed in more detail in the aforementioned references. The area of interest, in which the simulations are performed is an urban area with the central square and several surrounding buildings. The considered layout is based on the part of the Madrid grid, proposed by METIS society in Reference [32], and is shown in Figure 3 in Section 3. The area consists of 7 mm-Wave BSs and several buildings. The BSs in the area are mm-Wave BSs operating at 30 GHz carrier frequency able to create 32 different beams. Moreover, each BS includes a linear array of 32 antenna elements, which form the beams based on the phased array principle. The codebook for beam selection ensures the orthogonality of the individual beams and is based on the discrete Fourier transform matrix. The dataset considered in this work was simulated by creating a mobile UE, in which movement was defined by switching waypoints on the deployed map with an additional curvature. The UE measures the RSRP from all available beams every 0.36 s, which correspond 0.48 m mean distance between two measurements if the UE moves at 5 km per hour mean speed.

The raw dataset includes 1,342,701 samples, and 226 features, consisting of 224 RSRP measurements and 2 positioning features (*x* and *y* coordinates). In order to generate the required input arrays for each training step, 10 samples were removed from the beginning and the end of the dataset (leaving 1,342,681 sample indexes). Next, the samples were split between the training, validation and testing data. The 60−20−20 split resulted in 3 independent datasets, as summarized in Table 2.

The ray-tracing tool and generated data, as well as the evaluation of the results were realized in MATLAB environment. The data pre-processing, creation of ANN models, training and generation of results were done in Python version 3.7.4. using modules Tensorflow, Pickle, Numpy, statmodels, and matplotlib. The ANN models were trained on Lenovo ThinkPad notebook with Intel Core i7-8750H CPU with 32 GB RAM.

### 4.2. Cell-Level Mobility Results

In order to evaluate the cell-level mobility model presented in Section 4.3, we introduced different levels of uncertainty to the RSRP values used as input data to the considered models, 3GPP benchmark and proposed DL model. Additionally, we evaluated the “Max RSRP” model as the optimal solution for maintaining the highest possible RSRP at all times to show the theoretical achievable maximum on the considered data. All models were evaluated under the same conditions, as the only decision the models were performing was choosing the serving cell, while the selection of the strongest beam at the chosen cell was completed automatically.

**Figure 6:**
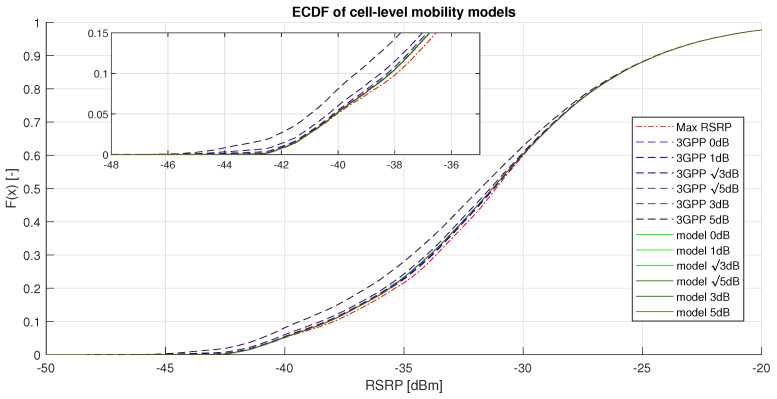
Experimental Cumulative Distribution Function (ECDF) of cell-level mobility models.

Figure 6 presents the distributions of serving RSRP of the considered models under different uncertainty conditions (representing shadowing, device-specific noise, interference, etc.). It displays the Experimental Cumulative Distribution Function (ECDF) of the given models, which visualizes the cumulative probability of the given variable. F(x)[−], represented by the *y*-axis, determines the probability of the serving RSRP being lower than *x* [dBm] (the value on the *x*-axis). The figure shows that the performance of the 3GPP model decreases with increasing measurement uncertainty level. The numerical results presenting the HO count and the mean RSRP level of the model are shown in Table 3, where it is directly visible that the number of HOs rapidly increases for the 3GPP model when the level of RSRP measurement uncertainty increases. Furthermore, with the 3GPP model, the number of HOs at 5 dB uncertainty level (32,864) compared to the 0 dB uncertainty level (1805) is more than 18 times higher. However, with the proposed model the number of HOs at 5 dB uncertainty level (1839) and at the 0 dB uncertainty level (1805) is almost identical, while still sustaining the mean RSRP levels.

The proposed solution was able to reduce the number of handovers by 94.4% compared to the benchmark model at uncertainty level 5 dB. The number of handovers at a high uncertainty level shows that the DL model proposed by authors successfully filters the uncertainty out of the measurements. The results show that, at 0 dB uncertainty level, the proposed model can approximate the baseline model perfectly, even at the independent dataset.

### 4.3. Beam-Level Mobility Results

**Figure 7:**
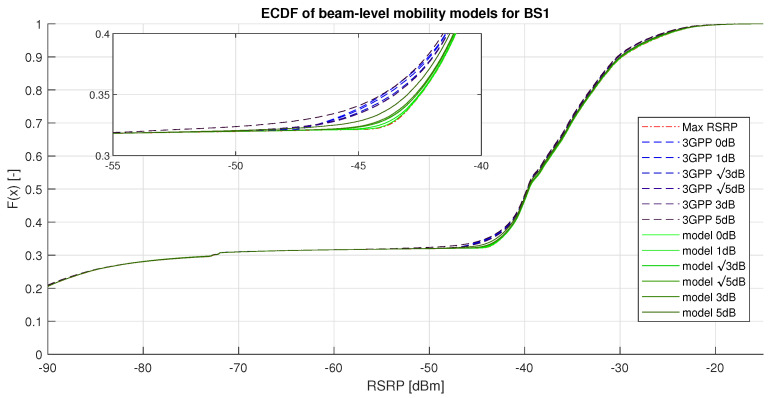
ECDF of beam-level mobility models on BS1 data.

The beam-level mobility model was evaluated on the independent testing dataset for data of BS1. The RSRP measurement data were introduced to varying levels of uncertainty, and the results of the evaluation are shown in Figure 7 and in Table 4. Additional model reflecting the maximum achievable RSRP called “Max RSRP” was included in the analysis. Figure 7 displays the ECDF of the serving RSRP for the considered models. There, F(x)[−] determines the probability of the serving RSRP being lower than *x* [dBm] (the value on the *x*-axis). The figure shows that the upper half of the distributions are almost identical for all models. This is caused by the fact that there is often only a single strong beam available, and all models were able to choose the serving beam successfully. The lower part of the figure is also similar for all models, as after losing line of sight (LOS), no strong beams become available, and non-line of sight (NLOS) measurements are significantly weaker. The detail in the figure shows the difference between the proposed model’s decision-making and the 3GPP one. There, the figure shows that the proposed solution has approximately 4% higher chance of achieving 44 dB or better signal strength than the 3GPP solution.

Table 4 presents the HO count and mean RSRP results comparing the considered models at the chosen uncertainty levels. The table shows that, at 0 dB uncertainty level, the 3GPP solution offers 2.5% lower number of HOs than the proposed solution with the trade-off of 0.50 dB lower mean RSRP. This difference is caused by the proposed model target, prioritizing the connection strength over the HO count. As stated above, beam-switching does not require network-level signaling and therefore affects the performance of the system much less than the inter-cell HO.

The results also show that the number of HOs of the 3GPP model increases much faster than the number of HOs of the proposed solution. At 5 dB uncertainty level, the 3GPP solution performed 2.7 times more HOs than at uncertainty of 0 dB, whereas with the proposed solution, the increase in HO count was only by 14%.

### 4.4. Discussion

In the following paragraphs we discuss the possible improvements to the training procedure of the models, challenges that may arise when applying the proposed solution (or any other ML model) to real-world data, and other available research questions.

Since the same model architecture is applied to each base station, it is possible to consider pre-training of the “blank” model on previously acquired data, thus applying transfer learning. This might significantly reduce the amount of data the model requires at the new location. The supervised pre-training is applied to numerous convolutional architectures, as the filters in the first layers detect basic shapes, which are universal in most cases (e.g., decreasing signal strength over time).

In this work, we assumed the distribution of the uncertainty to be Gaussian, perfectly uncorrelated in time. This may not be true in many cases when using the real-world data, as signal shadowing, UE antenna orientation or UE velocity change with limited time dynamics. Moreover, different types of UE have different equipment-specific characteristics, which creates additional data uncertainty. It is possible for NN to adapt to the colored noise in the measurements, but it has to be included in the training data and often requires more complex NN models.

Another challenge that may arise is the question of granularity of measurements. The simulation was set up, so the distances between the measurements were equidistant in the temporal region; therefore, the distances in space were reflecting the user’s speed. In the aperiodic CSI reporting scenario, the model will require additional information to predict the next serving beam or cell correctly.

This work considered the RSRP as a single value related to a user’s spatial position and assumed the environment to be constant in time. In reality, the RSRP also depends on the measured frequency band, as well as on the temporal information (changing weather, network load, time of year, etc.). We also considered only a single user scenario. Extending the solution to multiple users is one of the challenges of our future work and the most promising direction to address it is to build additional models, which can work in series with the currently proposed ones. This work’s NN models, while considering only a single user, are of low complexity. Therefore, stacking them in parallel (one for each user), while feeding their outputs to the common system promises the low-complexity, segmented, and elegant solution. Extending the proposed solution to reflect previously mentioned aspects is required before fully deploying it for the operation in the real-world.

## 5. Conclusions

In this work, the authors present a novel approach to mobility management in beamforming-based deployment for 5G NR networks. The proposed solution for both cell-level and beam-level mobility management model is based on ANN with multiple inputs and hybrid architecture, including both convolutional and dense layer blocks, and focuses on low-complexity and fast performance. The proposed models consider current, as well as past, RSRP measurements, in addition to current positioning information, as the inputs. The cell-level mobility management model also considers the current serving beam.

The scenario of interest was densely built urban area with an open area central square and different building sizes. The dataset was created using a 3GPP-specified ray-tracing simulator and included positioning information and RSRPs of all measured beams at each measurement instance.

The cell-level mobility model performed on the same level as the benchmark 3GPP solution when no uncertainty was present in the data. When increasing the uncertainty level, the performance of the 3GPP solution (regarding both serving cell RSRP and HO count) rapidly degraded, while the proposed model sustained both high RSRP and low HO count at all uncertainty levels. The deciding metric in case of cell-level mobility is low HO count, and the proposed solution achieved 94.4% less HOs than the 3GPP model at RSRP uncertainty level of 5 dB. The performance of the beam-level mobility management model was evaluated on BS1 data. The proposed solution was designed to always sustain the strongest possible beam and achieved 0.5 dB higher mean RSRP than the 3GPP model at uncertainty level 0 dB. The HO count of the proposed solution is higher at the 0 dB uncertainty level than that of the benchmark model and lower at uncertainty levels above $\sqrt{3}$ dB. Beam-level HOs do not have a significant impact on network performance, while sustaining high serving RSRP is required.

The work presents the high potential of DL systems in 5G mm-Wave networks. We demonstrate the drawbacks of reactive solutions, such as considered the 3GPP model, and show that the proposed NN solution can proactively handle both cell-level and beam-level mobility optimally at all considered uncertainty magnitudes.

## Figures and Tables

**Table 1:** Hysteresis margin effect on Reference Signal Received Power (RSRP) and handover (HO) count for BS1 data.

Hysteresis margin [dB]	0	1	$\sqrt{3}$	$\sqrt{5}$	3	5
HO count [-]	8629	8458	8363	8279	8182	8054
Mean RSRP [dBm]	−53.14	−53.20	−53.29	−53.39	−53.51	−53.64

**Table 2:** Train-validation-test split characteristics.

Dataset	Split Size	Num. of Samples	First Sample Index	Last Sample Index
Train	60	805,609	11	805,619
Validation	20	268,536	805,620	1,074,155
Test	20	268,536	1,074,156	1,342,691

**Table 3:** HO count results for cell-level mobility evaluating the performance of benchmark 3rd Generation Partnership Project (3GPP) model and proposed Deep Learning (DL) model with different levels of uncertainties present in the input measurements.

Model	3GPP						Proposed Model					
Uncertainty [dB]	0	1	$\sqrt{3}$	$\sqrt{5}$	3	5	0	1	$\sqrt{3}$	$\sqrt{5}$	3	5
HO count [-]	1805	2830	5665	9375	15,533	32,864	1805	2010	1783	1795	1884	1839
Mean RSRP [dBm]	−31.21	−31.18	−31.19	−31.23	−31.33	−31.74	−31.21	−31.20	−31.20	−31.20	−31.21	−31.21

**Table 4:** HO count results for beam-level mobility for BS1 evaluating the performance of benchmark 3GPP model and proposed DL model with different levels of uncertainties present in the input measurements.

Model	3GPP						Proposed Model					
Uncertainty [dB]	0	1	$\sqrt{3}$	$\sqrt{5}$	3	5	0	1	$\sqrt{3}$	$\sqrt{5}$	3	5
HO count [-]	8182	8276	8576	9256	11,192	22,195	8389	8706	8943	9378	9424	9513
Mean RSRP [dBm]	−53.51	−53.47	−53.42	−53.49	−53.49	−53.65	−52.98	−53.01	−53.03	−53.04	−53.07	−53.04
